# Exercise therapy for the management of osteoarthritis of the hip joint: a systematic review

**DOI:** 10.1186/ar2743

**Published:** 2009-06-25

**Authors:** Peter J McNair, Marion A Simmonds, Mark G Boocock, Peter J Larmer

**Affiliations:** 1 Health and Rehabilitation Research Centre, Auckland University of Technology, Private Bag 92006, Auckland 1020, New Zealand

## Abstract

**Introduction:**

Recent guidelines pertaining to exercise for individuals with osteoarthritis have been released. These guidelines have been based primarily on studies of knee-joint osteoarthritis. The current study was focused on the hip joint, which has different biomechanical features and risk factors for osteoarthritis and has received much less attention in the literature. The purpose was to conduct a systematic review of the literature to evaluate the exercise programs used in intervention studies focused solely on hip-joint osteoarthritis, to decide whether their exercise regimens met the new guidelines, and to determine the level of support for exercise-therapy interventions in the management of hip-joint osteoarthritis.

**Methods:**

A systematic literature search of 14 electronic databases was undertaken to identify interventions that used exercise therapy as a treatment modality for hip osteoarthritis. The quality of each article was critically appraised and graded according to standardized methodologic approaches. A 'pattern-of-evidence' approach was used to determine the overall level of evidence in support of exercise-therapy interventions for treating hip osteoarthritis.

**Results:**

More than 4,000 articles were identified, of which 338 were considered suitable for abstract review. Of these, only 6 intervention studies met the inclusion criteria. Few well-designed studies specifically investigated the use of exercise-therapy management on hip-joint osteoarthritis. Insufficient evidence was found to suggest that exercise therapy can be an effective short-term management approach for reducing pain levels, improving joint function and the quality of life.

**Conclusions:**

Limited information was available on which conclusions regarding the efficacy of exercise could be clearly based. No studies met the level of exercise recommended for individuals with osteoarthritis. High-quality trials are needed, and further consideration should be given to establishing the optimal exercises and exposure levels necessary for achieving long-term gains in the management of osteoarthritis of the hip.

## Introduction

Osteoarthritis (OA) is a major problem in modern society. In Western populations, the estimated prevalence for hip-joint OA is between 1% and 11% [1,2]. Treatments are typically directed at the management of symptoms, such as pain relief and improving function, with exercise therapy being commonly used as a treatment modality.

Recently, a Physical Activity Guidelines Advisory Committee report to the U.S. Department of Health and Human Services [3] provided guidelines concerning physical activity for those individuals with disabilities. This report made specific mention of exercise for those with OA, and the guidelines recommended that adults should get at least 150 minutes of moderate-intensity or 75 minutes of vigorous-intensity aerobic activity per week. Furthermore, it was recommended that they also participate in muscle-strengthening activities of moderate or high intensity on 2 or more days per week. These recommendations are very similar to those of the American College of Sports Medicine [4] that individuals aged between 50 and 64 years with chronic conditions such as arthritis need to undertake moderately intense cardiovascular exercise 30 minutes per day, 5 days per week or undertake vigorously intense cardiovascular exercise 20 minutes per day, 3 days per week, and undertake eight to 10 strength-training exercises (eight to 12 repetitions of each exercise) twice per week.

These guidelines seem rigorous, even for those who are able bodied, and whether they can be realistically achieved by those individuals with OA of the hip is questionable. Epidemiology data concerning physical-activity levels of individuals without OA support this suggestion. For instance, Macera *et al*. [5] examined whether U.S. adults were meeting physical-activity recommendations similar to those mentioned earlier, and reported that approximately 42% of men and 32% of women older than 65 years were participating at the appropriate levels. More recently, Ham *et al*. [6] reported that on any given day in the United States, only 29% of men and 22% of women aged between 40 and 75 years participate in physical activity for longer than 30 minutes, and this activity included a combination of sports, exercise, and recreational activities. Notably, these activities levels were decreased when individuals were overweight or obese, which is not uncommon in those with OA of the hip joint. Furthermore, given that individuals with OA are also often afflicted with considerable pain, loss of function, depression, and poor self-efficacy [7], one might not be surprised at their unwillingness or ability or both to participate in exercise of an intensity and duration recommended in the guidelines.

One method of investigating whether such levels of exercise are needed in individuals with OA of the hip is to examine intervention studies focused on this cohort to determine what levels of exercise have been required for notable decreases in pain and improvements in function and quality of life. Focusing such a study on the hip joint would be valuable, as reviews of OA have highlighted the very limited amount of data available to assess the efficacy of strengthening and aerobic exercise for those individuals with hip-joint OA [8-10]. Whether this reflects a dearth of good-quality studies or insufficient exercise programs remains to be determined.

Thus, the aim of this study was to conduct a systematic review of the literature to evaluate the exercise programs used in intervention studies focused solely on hip joint OA and to decide whether they met the recommendations of the guidelines highlighted earlier, and also to determine the efficacy of their exercise-therapy interventions for improving pain levels, function, and quality of life.

## Materials and methods

### Search

An initial search of the literature was undertaken by using a variety of sources, including textbooks, conference proceedings, and previous systematic or critical reviews from the published literature. From this initial search, an extensive keyword list was developed that included terms specific to exercise interventions and OA of the hip. These were hip, osteoarthritis, osteoarthritic, pain, function, quality of life, exercise, rehabilitation, physical therapy, physiotherapy, hydrotherapy, aquatic, strength(ening), resistance, aerobic, endurance, stretch(ing)(es), train(ing), protocols. An initial check of the keyword list was made against each of the subject headings from 14 electronic databases (AMED, Annual Reviews, Blackwell Synergy, CINAHL, EBM reviews (including Cochrane Reviews), EBSCO health databases (including MEDLINE), EMBASE, Expanded Academic ASAP, Index NZ, Lippincott 100, PEDro, ProQuest 5000, PsycINFO, Science Direct, and Sports Discus). The literature search was also supplemented with a review of the bibliographies of past review papers on exercise-therapy interventions, as well as the personal libraries of the contributing authors. When searching for past review articles, additional keywords were added to the main keyword list. These included "review", "critical", "meta" and "systematic". Two researchers carried out the literature search. The keyword list and all combinations of keywords were used uniformly by both researchers to ensure a standardized approach to the search procedure.

### Study selection

To be eligible for inclusion in the review, randomized controlled trials and quasi-experimental studies in which an intervention was compared with another or with a control group had to meet the following criteria. Studies were restricted to patients with hip OA solely (patients with a comorbidity of joint OA, i.e., knee arthritis were excluded). Diagnosis in studies was defined according to symptoms consistent with OA (e.g., restriction and pain on specific hip movements, stiffness in the morning no longer than an hour) and/or radiologic findings (with or without physical examination). Exercise therapy must have been used as an intervention with a corresponding control or a comparison intervention group. Exercise therapy was defined as activities such as strengthening, aerobic conditioning, stretching, endurance, hydrotherapy, or a combination of these that lasted for at least 3 weeks. The review was restricted to English-language publications.

No limitation was placed on the date of publication, and articles were retrieved to June 2008. Studies were excluded if they involved specific pre- or postoperative exercise therapy; however, studies that included subjects who were on waiting lists for surgery were acceptable.

### Data extraction

Two authors extracted data from the selected studies. These data were tabulated under the headings: study design, intervention, outcome measures, and main findings. The variables of interest were pain, function, and quality of life. Where possible, pre- and post- intervention means and standard deviations for the outcome measures were extracted, and effect sizes (ESs) were calculated [11]. Any ESs reported in the studies were also recorded.

### Internal validity of the studies

The appraisal and grading of intervention studies involved a modified version of the Cochrane Musculoskeletal Injuries Group (CMIG) scoring system [12]. The CMIG scoring system comprises of 13 separate questions graded between 0 and 2, covering aspects of study design and outcome measures. A final overall score (quality rating), of a possible 26, was awarded to each intervention article. Three reviewers (authors: MS, PL, and MB) were trained in the review and scoring protocols. Two reviewers scored each article independently, and if any discrepancies were found between the two reviewers, a third person reviewed the article so that a consensus could be reached.

### Data synthesis

Owing primarily to the expected heterogeneity in the variables of interest, statistical pooling of the data was not appropriate. Thus, to assess the overall findings a 'pattern of evidence' approach was used [13]. This approach considered the consistency of findings across studies, the design of the studies (e.g., RCT, pre- and post-design) and the quality level of the studies. These criteria allowed the categorization of evidence into four levels: strong, moderate, some, or insufficient [14] (see Table 1 for the definitions associated with these categories). A study was considered to be of low quality if it scored less than 14 of 26, medium quality if it scored more than 13 (50%) of 26, but less than 21 (80%) of 26, and of high quality if it scored equal to or more than 21 of 26. If fewer than 75% of studies reported the same trend in findings across each of the variables of interest (pain, function, and quality of life), then the findings for that variable were deemed inconsistent.

**Table 1:** Level of evidence for evaluating the efficacy of exercise therapy in the management of osteoarthritis of the hip

Level of evidence	Definition
Strong evidence	Generally consistent findings in multiple trials of high quality (QS = 21)
Moderate evidence	Findings in one high-quality study and one other medium-quality trial or by generally consistent findings in multiple trials of medium quality
Some evidence	Generally consistent findings in at least one trial of medium quality (QS > 13), and/or consistent findings in multiple low-quality trials
Insufficient evidence	Findings from one low-quality trial or generally inconsistent findings in multiple trials

QS = Quality rating.

## Results

### Studies included in the review

**Figure 1:**
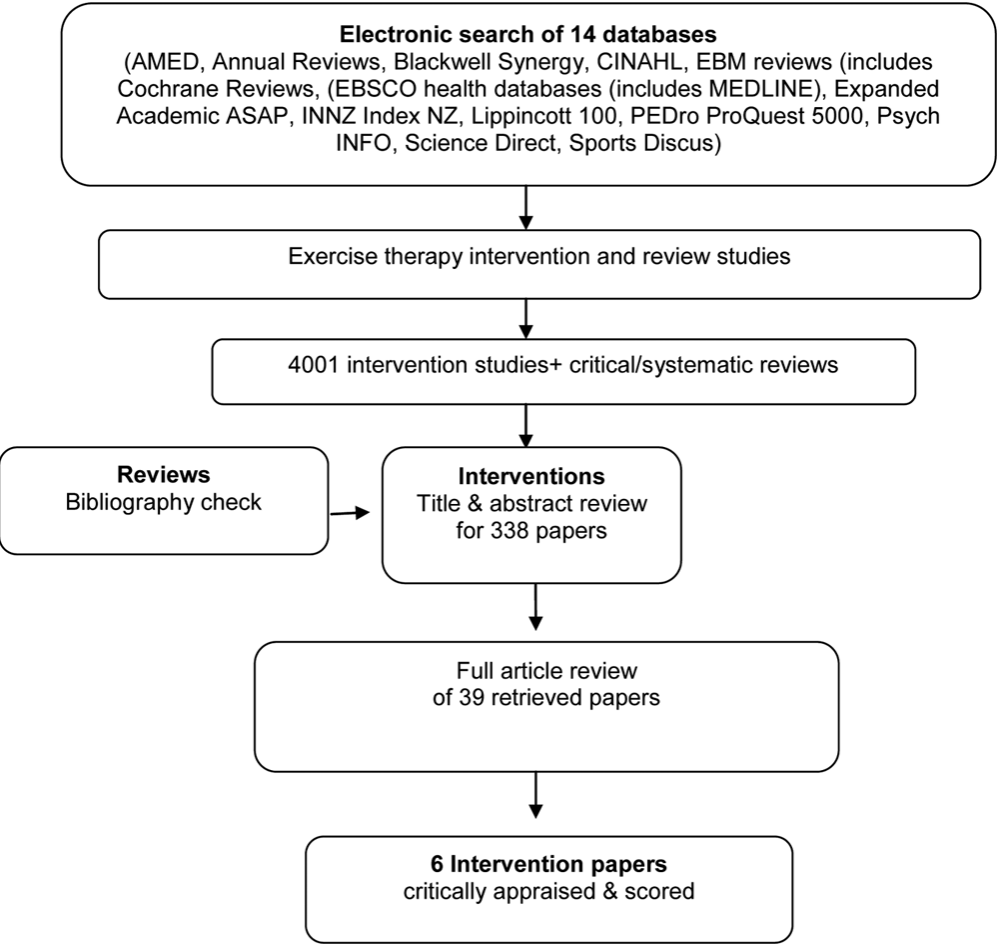
Flow chart of trial selection process.

From the initial literature search, 4,001 articles were identified, of which 338 intervention articles were considered suitable for abstract review. Thereafter, 39 articles received a full review, and from these articles, six intervention studies were considered to have met the inclusion criteria and were subject to critical appraisal and scoring (see Figure 1). The primary reasons for the rejection of articles were that studies did not separate data/results related to the subjects with hip-joint OA when subjects with hip and knee OA were used; and second, the intervention was not focused sufficiently on exercise. The information relating to each article included in the review, is shown in Table 2.

**Table 2:** Summary of intervention studies

Author	Design • Intervention • Control group • Recruitment • Diagnosis/Condition • Baseline pain levels	Intervention • Intervention category • Dosage • Exercises • Follow-up	Measures
Green *et al*. [20]	• Hydrotherapy and home exercise • Home exercise only • 47 subjects referred from specialist clinics (mean age, 66.8 years) • OA hip diagnosed with radiology (with approximately 75% of subjects moderate to severe). Hip pain ≥ 6 months. Normal ESR and negative rheumatoid factor • No baseline pain measures provided.	• Hydrotherapy and home exercise *vs*. home exercise only • Two groups of subjects: Hydrotherapy and home exercise: (24 subjects) home exercise 2× daily and hydrotherapy 2× per week for 6 week Home exercise only: (23 subjects): 2× daily for 6 weeks with compliance monitored • 3 mobility and 2 strengthening exercises; 10 repetitions progressing to 30 • Baseline measurements 3 times over 6 weeks before intervention, immediately after intervention, then follow-up at 6 weeks and 3 months	Pain VAS Hip function Gait parameters
Haslam [19]	• Acupuncture • Exercise therapy • 32 subjects referred from specialist clinics (> 39 years) • OA hip diagnosed with radiology, excluding RA, steroid injection, and hip surgery. Mean duration of symptoms was 6 and 9 years • No information provided concerning baseline pain levels	• Acupuncture *vs*. exercise therapy • Two groups of 16 subjects: Acupuncture: 25 minutes, 1× per week for 6 weeks Exercises and advice: baseline visit and 3-week check-up to correct exercises and progressed gently • 5 exercises (not described) • Measurements before and after intervention, then follow-up at 2 months	Self-reported pain and function Modified WOMAC questionnaire
Hoeksma *et al*. [15]	• Combined exercise therapy • Comparison intervention manual therapy • 109 subjects referred from specialist clinics (> 60 years) • Unilateral OA hip diagnosed by using American College of Rheumatology criteria (with approximately 80% of subjects moderate to severe). Hip symptoms ranged from 1 month to ≤ 10 years • Baseline mean pain level during walking was 29 and 34/100 within groups	• Exercise therapy *vs*. manual therapy • Two groups of 109 subjects: Exercise therapy: (53 subjects) 25 min 2× per week for 5 weeks, total of 9 individual sessions + home program Manual therapy: (56 subjects) 25 min 2× per week for 5 weeks total of 9 individual sessions (hip-joint stretches, manual traction, manipulation traction and education) • Strengthening with weights, endurance (treadmill or cycling), range of motion, stretches, balance, and education). • Measurements before and after intervention and then follow-up at 3 and 6 months	Pain VAS for pain at rest, on walking, and main complaint Pain subscale on HRQOL (SF-36) questionnaire Hip function Walking-speed parameters HRQOL (SF-36) subscales of physical function
Stener-Victorin *et al*. [18]	• Hydrotherapy and education • One control (education only) and one comparison intervention (electro-acupuncture and education) • 45 subjects referred from specialist clinics (> 42 years) • OA hip diagnosed by general practitioner with x-rays and pain consistent with OA • Baseline median pain level during loading was 37, 55, and 56/100 within groups	• Hydrotherapy *vs*. control *vs*. acupuncture • Three groups of 15 subjects: Hydrotherapy & education: 30 min, 2× per week for 5 weeks (10 sessions) Electro-acupuncture & education: 30 min, 2× per week for 5 weeks (10 sessions) Education only: 2-hr group session, 2× over 5 weeks. Included exercises undertaken once per day • 10 exercises (not described) to improve joint strength, stability, and range of motion • Measurements before and after intervention, then follow-up at 1, 3, and 6 months	Pain VAS for pain related to motion and loading, ache during day, ache during night Self-reported function Disability Rating Index Quality of life Global Self-rating Index
Sylvester [17]	• Hydrotherapy • Short-wave diathermy (SWD) and light exercises • 14 subjects referred from specialist clinics (> 49 years) • Not stated how OA hip was diagnosed Hip symptoms range from 2 to 8 years • Baseline median pain level was 78 and 83/100 within groups	• Hydrotherapy *vs*. comparison intervention • Two groups of 7 subjects: Hydrotherapy: 30 min, 2× per week for 6 weeks Short-wave diathermy and exercises similar to those of hydrotherapy group: 30 min, 2× per week for 6 weeks • Walking, leg swings, and mobility exercises • Measurements before and after intervention only	Pain VAS Self-reported function Oswestry Disability questionnaire Quality of life Philadelphia questionnaire
Tak *et al*. [16]	• Strengthening and health education • General medical practice • 109 subjects, community volunteers (> 55 years) • OA Hip diagnosed by general practitioner by using American College of Rheumatology criteria [35] • Baseline mean pain level was 38 and 42/100 within groups	• Strengthening and health education (ergonomic advice from occupational home visit, and dietary advice) *vs*. control • Two groups of 109 subjects: Strengthening and health program: (55 subjects) 1 hr 1× per week for 8 weeks Control: (54 subjects) self-initiated contact with their own GP • Strength training using fitness equipment; 2 levels of intensity: light and moderate; and a home exercise program • Measurements before and after intervention and then follow-up at 3 months	Pain VAS Pain subscale on Harris Hip Score (HHS) Self-reported hip function Groningen Activity Restriction Scale Hip function Time to perform 4 functional tasks (walking 20 m, stairs, timed up and go, toe reaching) Quality of life Quality of life VAS Health-Related Quality of Life Questionnaire (HRQOL)

### Quality

The scores related to the quality of the articles (QS) varied from 6 to 21 of 26. One article [15] attained an 80% score (21 of 26), whereas a second article [16] achieved a 60% score (16 of 26). All others were at 50% or less. The key elements associated with the quality of each article are presented in Table 3. It shows that aspects related to blinding of subjects and treatment providers were the key issues that were not addressed well.

**Table 3:** The quality-rating scores of articles

	Green [20]	Haslam [19]	Hoeksma [15]	Stener-Victorin [18]	Sylvester [17]	Tak [16]
A: Concealed allocation	1	0	2	0	1	1
B: Intention to treat	1	0	2	0	0	2
C: Blinded assessors	1	0	2	0	1	2
D: Comparable groups	1	1	2	0	0	2
E: Blinded subjects	0	0	0	0	0	0
F: Blinded treatment providers	0	0	0	0	0	0
G: Identical care programmes	1	0	2	0	1	0
H: Inclusion criteria	1	2	2	2	0	2
I: Relevant diagnostic criteria	2	1	2	1	0	1
J: Outcomes defined	1	2	2	2	1	2
K: Diagnostic tests useful	1	0	1	1	0	1
L: Duration of surveillance	1	0	2	1	0	1
M: Intervention practical	2	2	2	2	2	2

Total	13	8	21	9	6	16

A. Was the assigned treatment adequately concealed before allocation?

B. Were the outcomes of patients who withdrew described and included in the analysis?

C. Were the outcome assessors blinded to treatment status?

D. Were the treatment and control groups comparable at entry?

E. Were the subjects blind to assignment status after allocation?

F. Were the treatment providers blind to assignment status?

G. Were care programs, other than the trial options, identical?

H. Were the inclusion and exclusion criteria clearly defined?

I. Are the diagnostic criteria used relevant?

J. Were the outcome measures used clearly defined?

K. Were diagnostic tests used in outcome assessment clinically useful?

L. Was the duration of surveillance clinically appropriate, with active and systematic follow-up?

M. Was there practical relevance of the intervention?

### Participants

Across all studies, 356 subjects were involved. Within and across studies, the number of subjects participating in intervention and control groups ranged from 7 to 56, with three of the six studies having fewer than 17 subjects per group. Patients were recruited primarily from specialist clinics (N = 247), but also included community volunteers (n = 109). The criteria for inclusion were varied and included the diagnostic guidelines of the American College of Rheumatology, radiology, and measures of pain. Subjects in some studies were on hip-replacement waiting lists, but none of the studies reviewed had focused their programs on preoperative exercise specifically in preparation for surgery. The mean age of subjects varied from 66 to 72 years, with subjects aged from 39 to 86 years. Across studies, the most commonly presented variable that provided a measure of disease severity was pain measured by a visual analogue scale (VAS). This ranged from 29 to 83 of 100, the highest values being in groups in Sylvester [17] (78 and 83 of 100). Other scores were all less than 60.

### Outcomes measures

The primary outcome measures used to evaluate the efficacy of each intervention varied between articles and were grouped into self-reported pain, hip function (self-reported or performance based), and quality of life. Examples of self-reported pain included the VAS; the pain subscale of the Harris Hip Score; and/or the pain subscale of the Health Related Quality of Life short-form 36 (HRQOL SF-36) questionnaire. Self-reported functional measures included the Harris Hip Score, the Western Ontario and McMaster Universities Osteoarthritis Index (WOMAC), the Groningen Activity Restriction Scale, or the Disability Rating Index questionnaire. Measures of function included performance tasks such as the 'timed up and go' test. Quality of life was assessed by HRQOL SF-36 questionnaire, Sickness Impact Profile questionnaire, Philadelphia questionnaire, Quality of Life VAS, or the Global Self-rating Index. Some studies included impairment measures such as strength and range of movement, but these were not examined in the current review.

### Interventions

The interventions included (a) hydrotherapy, which was primarily of low intensity and involved walking, leg swinging, and mobility exercises; (b) land-based swinging, mobility, and stretching exercises; (c) strengthening exercises using fitness equipment or isometric contractions; (d) gait exercises; and (e) balance exercises. In many instances, combinations of these exercises were used. All but one study included groups who were supervised at a rehabilitation center, and a number of studies compared these groups with home-based exercise groups. Across studies, the reported duration of each exercise session ranged from 25 to 60 minutes, and these were held 1 to 7 days per week over a 5 to 8 week period. In some studies, the duration of exercise was determined according to the number of repetitions undertaken. The progression of exercise was not well defined in the majority of studies and included terms such as 'gentle', 'low', or 'moderate' without definitions, or was based on repetitions completed, and these varied between 10 and 30.

### Key findings

#### Pain

The two studies that scored highest in quality (QS) used land-based exercise programs. Hoeskma *et al*. [15] (QS, 21) compared an extensive exercise program with a manual therapy program, with both groups receiving patient education. The findings showed that bodily pain, as measured by the SF-36 subscale, was not different across groups. However, pain at rest (VAS score) showed a significant difference in favor of the manual therapy group immediately after the intervention (ES, 0.5) and at a 17-week follow-up (ES, 0.3). Pain during walking had a similar response (ES, 0.5) that extended to a 29-week follow-up. Tak *et al*. [16] (QS, 16), who compared a supervised strengthening program with a standard-care control group reported a significant improvement in pain levels as measured by the pain component of the Harris Hip Score (ES, 0.51) immediately after the intervention program and at a 3-month follow-up (ES, 0.38). These effects were less when measured with a VAS (ES, 0.00 after treatment and 0.17 at a 3-month follow-up).

In studies that had quality scores of 50% or less, Sylvester [17] (QS, 6) examined hydrotherapy compared with short-wave diathermy with light land-based exercise and reported decreased pain in both groups; however, no difference was found in effects across groups. Sterner-Victorin *et al*. [18] (QS, 9) used a similar prescription of hydrotherapy and noted that pain related to motion and loading activities was not different across hydrotherapy, electro-acupuncture and education-only groups at any assessment points. However, these authors reported a delayed effect for the hydrotherapy group, who experienced less pain during the day and night at a 1-month follow-up. In a study by Haslam [19], acupuncture was compared with exercise; however, pain and function levels were combined by using the WOMAC score. The findings showed that the acupuncture group had a significantly greater improvement in WOMAC scores compared with the home-exercise group immediately after treatment (ES, 0.62), although it should be noted that considerable drop-outs were found in the exercise group (44%).

#### Function

Hoeskma *et al*. [15] (QS, 21) reported that immediately after treatment, the SF-36 (role physical function) showed a significant difference in favor of exercise (ES, 0.4); however, the SF-36 (physical function subscale) showed no significant difference across manual therapy and exercise groups. For walking speed, significant differences were observed in favor of the manual therapy group immediately after treatment (ES, 0.3) and at 3-month follow-up (ES, 0.5). Tak *et al*. [16] (QS, 16) reported that performance measures related to function were not improved across strength-training and standard-care groups immediately after treatment. At the 3-month follow-up, the only significant change favoring the exercise group across four performance tests was the timed up-and-go test. Nonsignificant changes were also noted for self-reported function problems measured by the Groningen Activity Restriction Scale. In lesser-quality studies, Sylvester [17] (QS, 6) showed that a hydrotherapy group improved in function to a greater extent compared with the land-based exercise group. Green *et al*. [20] (QS, 13), whose study focused on home exercise with the addition of hydrotherapy, reported that tasks related to function were notably improved in both groups, with no difference across groups. However, no data were provided to support these comments. Sterner-Victorin *et al*. [18] (QS, 9) reported a delayed effect for a hydrotherapy group who improved in function compared with the education-only group at 1 month after exercise. Three months after treatment was completed, function was significantly greater in the hydrotherapy and electro-acupuncture groups compared with the education-only group.

#### Quality of life

Tak *et al*. [16] (QS, 16) and Sylvester [17] (QS, 6) found no changes in this variable, whereas Stener-Victorin [18] (QS, 9) reported that at 1 month after intervention, it was significantly improved in hydrotherapy and electro-acupuncture groups compared with an education-only group; however, by 3 months, the improvement remained in the electro-acupuncture group only.

### Evidence classification

Because of the lack of quality in studies and inconsistent findings across studies, the level of evidence in support of exercise as an effective treatment for hip-joint OA was limited. 'Insufficient evidence' (see Table 1 for definitions) was found to support exercise as a treatment for decreasing pain, improving function, or enhancing quality of life.

## Discussion

This review identified six trials that investigated the efficacy of exercise-therapy programs specific to patients with hip OA. It was apparent that very few articles addressed the effects of exercise on hip OA specifically. A previous review by Van Baar *et al*. [10] also highlighted this point, and it seems unusual that researchers have not pursued this area of research in the intervening years. Some studies have included hip and knee OA subjects in exercise interventions, but data related to the findings for hip and knee joint were not provided separately, a comment also made by Christie *et al*. [21].

Across the studies, wide-ranging levels of quality were noted, with only one study rated as high quality. Many studies had relatively small subject numbers, and in most studies, different treatments were compared without a control group. The study with the closest to what might be termed a control group was that of Tak *et al*. [16], whose control group was self-initiated contact with the subject's general practitioner. In some studies, although exercise was the predominant component of a program, other components such as education and advice were included.

The current review focused on three outcomes areas: pain, function, and quality of life. Despite this focus, a problem that emerged in the analysis was the numerous measures that fall within each of these areas. Within some of the studies assessed, the results for a particular variable (e.g., function) were different depending on the measurement used. Such differences highlighted the need to adopt internationally agreed key outcome measures.

There was 'insufficient evidence' to support exercise as a treatment to decrease pain. This result was in contrast to reviews by Van Barr *et al*. [10], Fransen [22], and Pisters *et al*. [23], which reported small to moderate effect sizes for exercise therapy decreasing pain associated with OA primarily at the knee joint.

'Insufficient evidence' was found for promoting exercise as a treatment to improve function. Reviews [10,22] focusing on knee-joint and/or a combination of knee and hip OA indicated only small effects arising from exercise programs, and a recent review by Pisters [23] noted contrasting findings across studies.

The current study also found little evidence to support exercise improving the quality of life. Similar findings were noted by Brosseau *et al*. [24], who commented that this finding may reflect the relatively short interval over which aerobic exercise programs are undertaken. In contrast, the same research team [25] reported that programs focusing on strengthening can be beneficial to quality of life, at least in the short term. Until recently [26], no quality-of-life measure has been developed specifically for OA. Hence the ability to see change (responsiveness) in this variable may have been limited by the content of questionnaires used.

Irrespective of the methodological issues associated with studies, the lack of notable improvements in the variables of interest may reflect the limited amount of exercise undertaken in studies. No studies met the levels set out in the aforementioned U.S. guidelines. Across all studies, the overall volume of exercise (duration per session and number of sessions per week) was well below the recommended levels. A key point in the guidelines concerns the intensity of exercise required. In this regard, information provided by authors in the current review was very limited. Often, the prescriptions of sets and repetitions for exercises were not provided in sufficient detail to indicate their merits, or the prescription was clearly insufficient to induce notable improvements in performance. Progression is a fundamental requirement of successful exercise programs [27]. In regard to individuals with arthritis, Petrella and Bartha [28] found greater improvements in pain levels and physical performance in participants who followed a progressive exercise program compared with those who did not. In the articles reviewed, often a lack of information was noted concerning how the training regimens progressed throughout their duration. In some studies, progression was implemented through increasing the number of repetitions of an exercise, not the intensity or load, which will lead to limited improvements, particularly in regard to strength and power.

Due to the limited number of studies that compared different types of exercise, no conclusions could be drawn as to whether one type was more beneficial than others. Similarly, other reviews [10,22,24,25] could not find evidence in support of a particular exercise therapy for the treatment of knee and/or hip OA. It may be that the lack of differences reflects the broad focus of some exercise programs. Attempting to address pain, range-of-motion, strength, mobility, and flexibility, as well as to incorporate education and gait training in 25- to 40-minute sessions over a 3 to 6 week period is likely to limit improvements in any one area. The work of Trudelle-Jackson and Smith [29] provides some evidence for a more-specific focus within exercise programs. Furthermore, as suggested by Van Baar *et al*. [10] and adopted by Hoeksma [15], it may be that targeting the individual's specific needs is a solution. However, if researchers take this pathway, it is important that authors provide descriptions of the criteria that led them to focus on a specific type of exercise and also provide the training parameters and improvements that occurred for those participants.

None of the studies assessed focused on cardiovascular fitness or provided a sufficient program to initiate notable improvements in this area, yet the importance of undertaking aerobic exercise for cardiovascular health is highlighted in the guidelines. A study [30] examining the cardiovascular fitness of those with OA showed peak VO_2 _consumption to be between 55% and 70% of matched subjects without OA. A lack of cardiovascular fitness has also been linked to comorbidities such as coronary heart disease [31]; therefore, it would beneficial for future research to target this aspect of fitness. Furthermore, as findings [32] suggest that individuals with low fitness levels who are having surgery are at more risk of having complications and mortality, effective cardiovascular programs would be of particular benefit to those individuals with arthritis who are facing a joint replacement.

Van Barr *et al*. [10] commented that a long-term follow-up often reveals a limited ability of exercise to maintain levels of function. This is not surprising. Unless subjects are specifically instructed to continue exercising, then a 'detraining' effect will become apparent [33,34]. In the studies examined in the current review, five involved follow-up assessments. However, only Green *et al*. [20] and Haslam [19] indicated that they instructed patients to continue exercising at home between the end of the formal training period and time of follow-up, but neither of these studies provided information concerning how much exercise subjects undertook during the time prior to the follow-up. Thus, the information obtained from these studies at follow-up has very limited value. Knowing when to institute "booster" sessions of exercise is an important area for future research that was highlighted recently by Pisters *et al*. [23].

Limitations existed in the current review. A meta-analysis was not performed because of the large variability of study designs, general poor quality of studies, and the lack of clearly defined similar dependent variables. Whereas the review included those studies using well-documented questionnaires and performance tests for outcomes, the validity and reliability of these measures could not always be determined. Unpublished studies, conference proceedings, reports, and Ph.D. theses were not reviewed. Reviewers were not blinded to authors or affiliations of published articles, and finally, the studies were restricted to those written in English.

## Conclusions

Few well-designed studies have specifically investigated the management of hip OA through the use of exercise therapy, despite evidence as to its potential benefits for the management of knee OA. Based on the studies included in this review, insufficient evidence was found to suggest that exercise therapy alone can be an effective short-term management approach for reducing pain levels, function, and quality of life. Furthermore, in respect to intensity, volume, and progression, it was apparent that exercise programs in the studies examined did not meet the current recommendations. Consideration should be given to establishing the optimal exercises and exposure levels necessary for achieving long-term gains in the management of OA of the hip.

## Abbreviations

AMED: Allied and Complementary Medicine Database; CINAHL: Cumulative Index to Nursing and Allied Health Literature; CMIG: Cochrane Musculoskeletal Injuries Group; EBM: evidence-based medicine; EBSCO: Elton B. Stephens Company; EF: effect size; EMBASE: Excerpta Medica Database; HRQOL SF-36: Health-related quality of life, short form 36; OA: osteoarthritis; PEDro: physiotherapy evidence database; PsycINFO: abstract database of psychological literature; VAS: visual analogue scale; VO_2: _the total amount of oxygen that the body needs and takes in; WOMAC: Western Ontario and McMaster Osteoarthritis Index.

## Competing interests

The authors declare that they have no competing interests.

## Authors' contributions

Peter McNair participated in the design of the study, review of findings, and wrote the final manuscript. Marian Simmonds participated in the design of the study, managed and undertook the search and critique of articles, and was involved in the writing of the manuscript. Mark Boocock and Peter Larmer critiqued articles, contributed to the interpretation of the findings, and participated in the writing of the manuscript.
